# Phytochemical Analysis and Antimicrobial Activity of *Myrcia tomentosa* (Aubl.) DC. Leaves

**DOI:** 10.3390/molecules22071100

**Published:** 2017-07-04

**Authors:** Fabyola Amaral da Silva Sa, Joelma Abadia Marciano de Paula, Pierre Alexandre dos Santos, Leandra de Almeida Ribeiro Oliveira, Gerlon de Almeida Ribeiro Oliveira, Luciano Morais Liao, Jose Realino de Paula, Maria do Rosario Rodrigues Silva

**Affiliations:** 1 Institute of Tropical Pathology and Public Health, Federal University of Goias, Goiânia 74605-050, Brazil; fabyola.sa@gmail.com; 2 Unit of Exact and Technologic Sciences, Goias State University, Anápolis 75132-400, Brazil; joelmapaula@uol.com.br; 3 Faculty of Pharmacy, Federal University of Goias, Goiânia 74605-170, Brazil; pierre@ufg.br (P.A.d.S.); leandra-almeida@hotmail.com (L.d.A.R.O.); 4 Chemistry Institute, Federal University of Goiás, Goiânia 74690-900, Brazil; gerlonribeiro@hotmail.com (G.d.A.R.O.); lucianoliao@ufg.br (L.M.L.)

**Keywords:** myrtaceae, antifungal activity, avicularin, *Candida* sp., phytochemistry, flavonoid

## Abstract

This work describes the isolation and structural elucidation of compounds from the leaves of *Myrcia tomentosa* (Aubl.) DC. (goiaba-brava) and evaluates the antimicrobial activity of the crude extract, fractions and isolated compounds against bacteria and fungi. Column chromatography was used to fractionate and purify the extract of the *M. tomentosa* leaves and the chemical structures of the compounds were determined using spectroscopic techniques. The antibacterial and antifungal activities were assessed using the broth microdilution method. The phytochemical investigation isolated 11 compounds: α-bisabolol, α-bisabolol oxide B, α-cadinol, β-sitosterol, *n*-pentacosane, *n*-tetracosane, quercetin, kaempferol, avicularin, juglanin and guaijaverin. The crude ethanolic extract and its fractions were tested against 15 bacteria and 9 yeasts. The crude extract inhibited the in vitro growth of yeasts at concentration of 4 to 32 μg/mL. The hexane, dichloromethane, ethyl acetate and aqueous fractions inhibited *Candida* sp. at concentrations of 4 to 256 μg/mL, whereas the *Cryptococcus* sp. isolates were inhibited only by the hexane and dichloromethane fractions in minimal inhibitory concentrations (MICs) at 16 to 64 μg/mL. The flavonoid quercetin-3-*O*-α-arabinofuranose (avicularin) was the most active compound, inhibiting *Candida* species in concentrations of 2 to 32 μg/mL. The MIC values suggest potential activity of this plant species against yeast.

## 1. Introduction

Infectious diseases are of great interest in the scientific community because some microorganisms cause severe morbidity and can be lethal. Plant species are a potential reservoir for the discovery of new drugs [1,2,3].

Among the plants of the Brazilian Cerrado, the Myrtaceae family has a great representation, and several species are used as ornaments, wood, food and medicines [4,5,6]. *Myrcia tomentosa* (Aubl.) DC. is a species of the Myrtaceae family and popularly known as “goiaba-brava”. It is a species native from the Brazilian Cerrado and can be found from Panama to the southeast of Brazil [7,8]. Despite its frequent citation in floristic or phytosociological surveys [4,9,10], *M. tomentosa* is underreported in pharmacognostic or phytochemical studies. Several biological activities have been described for the species of this genus, such as the inhibition of thyroid peroxidase, anti-obesity, hypolipidemic, hypoglycemic, antimicrobial, antioxidant, antifungal, anti-inflammatory, anti-nociceptive and hepatoprotective activities [11,12,13,14,15,16,17]. These activities are often attributed to the presence of secondary metabolites, such as their essential oils, but with few properties related to their non-volatile compounds [6].

Because of the scarcity of studies about *M. tomentosa*, the aim of this work is to realize the first bioassay-guided isolation of the extract and structural elucidation of the compounds, so as to verify the antimicrobial activity of these extracts and compounds against certain bacterial and fungal pathogens.

## 2. Results and Discussion

**Figure 1:**
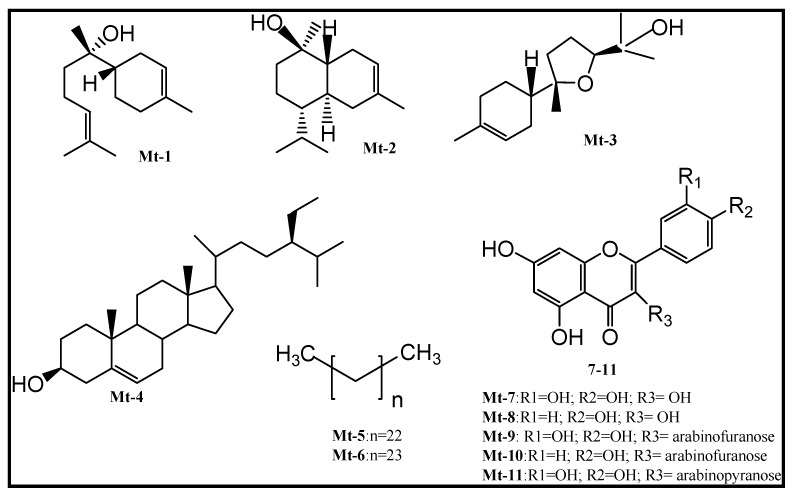
Chemical structures of compounds isolated from the leaves of *Myrcia tomentosa*. (**Mt-1**) α-bisabolol. (**Mt-2**) α-cadinol. (**Mt-3**) α-bisabolol oxide B. (**Mt-4**) β-sitosterol. (**Mt-5**) *n*-tetracosane. (**Mt-6**) *n*-pentacosane. (**Mt-7**) Quercetin. (**Mt-8**) Kaempferol. (**Mt-9**) Avicularin. (**Mt-10**) Juglanin. (**Mt-11**) Guaijaverin.

The chemical structures of the compounds were elucidated using Nuclear Magnetic Resonance (NMR) data with 2D experiments Heteronuclear Single Quantum Coherence (HSQC) and Heteronuclear Multiple Bond Correlation (HMBC), Gas Chromatography/Mass Spectrometry (GC/MS) and comparison with the literature (copies of the original spectra can be obtained from the corresponding author). The compounds isolated from the leaves of *M. tomentosa* (Figure 1) were identified as α-bisabolol (**Mt-1**) [18], α-bisabolol oxide B (**Mt-2**) [19], α-cadinol (**Mt-3**) [19], β-sitosterol (**Mt-4**) [20,21], *n*-pentacosane (**Mt-5**) [19], *n*-tetracosane (**Mt-6**) [19], quercetin (**Mt-7**) [22], kaempferol (**Mt-8**) [23], avicularin (**Mt-9**) [22,24], juglanin (**Mt-10**) [25] and guaijaverin (**Mt-11**) [26].

The sesquiterpenes (α-bisabolol, bisabolol B oxide and α-cadinol), the hydrocarbons (*n*-pentacosane and *n*-tetracosane), the steroid (β-sitosterol) and the flavonoids (quercetin, kaempferol, guaijaverin) were isolated and identified for the first time in this species.

The study biomonitored the fractions of *M. tomentosa* which allowed the isolation of substances that were responsible for the antimicrobial activity of this plant species. The results showed the antimicrobial activity by screening the crude extract and its fractions.

According to Holetz et al. [27] and other authors, such as Ayres et al. [28] and Regasini et al. [29], the MIC values below 100 μg/mL have good antimicrobial activity; an MIC from 100 to 500 μg/mL represents moderate antimicrobial activity; an MIC from 500 to 1000 μg/mL represents weak activity; an MIC above 1000 μg/mL suggests that the substance is inactive.

The antimicrobial activity against bacteria and yeasts using the crude ethanolic extract and fractions of *M. tomentosa* showed that the crude extract was inactive or weakly active for most tested bacteria; however, for the yeasts of the genus *Candida* and *Cryptococcus neoformans* species complex, the MIC range was 4–32 μg/mL. For the fractions, the antibacterial activity was moderate for some Gram-positive bacteria, and the ethyl acetate and aqueous fractions showed MICs of 125–500 μg/mL against *P. aeruginosa* (Table 1). Although the constituents of this plant, such as sesquiterpens, exhibit well-known antibacterial activity, the fractions isolated from the *M. tomentosa* leaves showed poor activity against bacteria.

The MIC values ranged of 4 to >1024 μg/mL against the studied yeasts. The polar fractions ethyl acetate fraction (EAF) and aqueous fraction (AF) and non-polar fractions hexane fraction (HF) and dichloromethane fraction (DF) were effective against *Candida* with low MIC values, which ranged from 4 to 256 μg/mL. *Cryptococcus* neoformans species complex was particularly inhibited by the non-polar fractions (HF and DF) of the leaf extract, with MIC values ranging of 16 to 64 μg/mL as shown in Table 1.

The main compounds obtained from HF and DF were identified as isolated sesquiterpenes (α-bisabolol, α-bisabolol oxide B and α-cadinol) or a mixture of these sesquiterpenes. Terpenes are a class of secondary metabolites with important functions in the interaction between a plant and its environment, as frequently implied in the defensive functions against herbivores and pathogens in the species of the family Myrtaceae [30]. In this study, the identified sesquiterpenes (α-bisabolol, bisabolol B oxide and α-cadinol), which were earlier reported on for their antimicrobial activity [31,32,33,34], were inactive in tested concentration against *Candida* sp. and showed onyl moderate activity (128 μg/mL) in the mixture. These results suggest that these sesquiterpenes partially contributed to the antifungal activity of this fraction, but also that other compounds are necessary for the fractions to exhibit good activity.

The compounds and mixtures of compounds of this plant evaluated against yeasts of the genus *Candida*, are showed in Table 1 and Table 2. Interesting results were obtained with avicularin isolated from polar fraction ethyl acetate. This substance showed a good antifungal activity (2–32 μg/mL) for all *Candida* strains; thus, it is mainly responsible for the activity of the fraction and probably the crude extract.

The flavonoid guaijaverin showed moderate activity for 55.5% of isolates and the juglanin showed similar activity for 44.4% of the isolates. Similar results were found by Metwally et al. [35] that reported good activity of avicularin and guajaverin against *C. albicans*.

The flavonoids and mixture flavonoids of this plant evaluated against yeasts of the genus *Candida* are showed in Table 2.

Several researchers, such as Kuete [36] and Martins et al. [37] associated the antimicrobial activity of aromatic plants with phenolic compounds. These compounds are mainly induced by fungal membrane damage with a consequent increase in cellular permeability [38]. Salazar-Aranda et al. [39] also suggested a structure-activity relationship where the hydroxylation pattern of the B or C ring of the flavonoid can determine its degree of antifungal activity.

Additionally, the results observed by Holetz et al. [27], Domingues et al. [40], Paula et al. [41] and Correia et al. [42] showed good activity against yeasts of other species of the Myrtaceae family, suggesting that compounds of *M. tomentosa*, as avicularin can be used as a potential antifungal.

In conclusion, the phytochemical analysis of *M. tomentosa* amplifies the chemical knowledge of the genus since the reported studies are mainly related to essential oils. In addition, the results of this study prove the antifungal activity of the ethanolic extract of *M. tomentosa* and its fractions and show that the flavonoid quercetin-3-*O*-α-arabinofuranose (avicularin) is mainly responsible for this biological activity and a potential source of new antifungal alternatives.

## 3. Materials and Methods

### 3.1. Plant Material

Leaves of three specimens of *M. tomentosa* were collected in August 2008 in Hidrolândia-GO, Brazil (16°53′59.4″ S 49°13′29.4″ W) and identified by J. R. de Paula. A voucher specimen was deposited in the Herbarium of the Federal University of Goiás under code number 41318.

### 3.2. General Procedures

The ^1^H and ^13^C one-dimensional and two-dimensional NMR spectra were obtained in deuterated chloroform (CDCl_3_) or deuterated methanol (CD_3_OD) on a Bruker Avance 500 MHz instrument (500 MHz for ^1^H and 125 MHz for ^13^C-NMR). The chemical shifts are expressed in δ values (ppm) with tetramethylsilane (TMS, δ = 0.0 ppm) as an internal reference. The coupling constants (J) were measured in Hertz (Hz). To process and analyze the spectra, the TopSpin ACD/Labs 12.0 programs were used.

The gas chromatography coupled to mass spectrometry (GC/MS) was performed on the chromatograph QP2010 (Shimadzu), which was equipped with a DB-5MS capillary column (30 m × 0.25 mm × 0.25 mM) using helium as the carrier gas. The injection volume was 1 µL, and the ionization energy was 70 eV. The parameters in the identification of chemical constituents are the presence of the molecular ion peak, basic peak, visual comparison with the spectra provided by the specter equipment [19], and fragmentation pattern in relation to the described mass spectra in the literature.

High-performance liquid chromatography (HPLC) was performed for the polar fractions using a Waters instrument e2596 with a quaternary pump, the diode array detector (DAD) 2998 and 2.0 Enpower data-processing system. The column was a Zorbax XDB C18 column (25 cm × 4.6 mm × 5 um), the flow was 1 mL/min, the temperature was 25 °C, and the injection volume was 10 µL. The mobile phase consisted of methanol and acidified water with 2% glacial acetic acid in different proportions. The samples were pre-filtered using a 0.45-µM Millex^®^ membrane (Millipore, Cork, Ireland) and a mobile-phase PVDF membrane of 0.45 micrometre (Millipore, Cork, Ireland).

The semipurified polar samples were submitted to a Sepacore preparative chromatograph (Buchi) with the peristaltic pump model C-615. A Sepacore^®^ C-18 column (9 cm × 10 mm) with a flow rate of 10 mL/min was used. The samples were previously filtered through a 0.45-μm Millex^®^ membrane (Millipore, Cork, Ireland) and chromatographed with the mobile phase, which consisted of methanol and purified water in different proportions.

The fractionations in chromatographic columns (CC) were performed using silica gel 60 (0.063–0.200 mm/70–230 mesh ASTM) (Macherey-Nagel). For the analytical thin-layer chromatography (TLC), silica gel plates were used (G60 F_254_ (Vetec)). After the solvent evaporated, the plates were observed under UV light at 254/365 nm, developed with a vanillin sulfuric acid solution and heated or developed with 2-aminoethyl diphenylborinate (NP) in a methanolic solution.

### 3.3. Extraction and Purification

The air-dried and powdered leaves of *M. tomentosa* (50 g) were extracted with 95% ethanol by maceration (1:5 *w*/*v*) at room temperature. The crude ethanolic extract was filtered and concentrated on a rotary evaporator at a temperature below 40 °C. Fifty grams of dried extract were solubilized in 200 mL MeOH/H_2_O (7:3) and subjected to a liquid/liquid extraction with solvents of increasing polarity (hexane, dichloromethane, and ethyl acetate). These fractions were concentrated on a rotary evaporator at 40 °C and maintained at the exhaust hood until the solvent was completely removed. The MeOH/H_2_O residual was lyophilized, which resulted in an aqueous fraction. The resulting fractions were named hexane fraction (HF), dichloromethane fraction (DF), ethyl acetate fraction (EAF) and aqueous fraction (AF).

A mass of 2.5 g of HF was fractionated by column chromatography with silica gel (1:40), which was eluted with hexane (100%) Hex-EtOAc (2–100%) and EtOAc-MeOH (1:1). Seventy-five (75) fractions were collected and analyzed by TLC after the solvent evaporated using the mixture Hex-EtOAc (10–40%). Based on the retention factors (*R*_f_) of the bands under 254/365 nm UV light, the development with vanillin sulfuric acid and subsequent heating of the solution, the fractions were 16 new pooled fractions: HF-1 to HF-16. The HF-2 and HF-3 fractions were rechromatographed in a silica eluted gel column in the isocratic mode with Hex-EtOAc (8:2), which resulted in **Mt-1** (278 mg). The HF-4 and HF-5 fractions were pooled and rechromatographed in a silica eluted gel column in the isocratic mode with Hex-EtOAc (8:2), which resulted in **Mt-1** (61 mg) and **Mt-2** (6 mg). The subfractions HF-4.3, HF-4.4 and HF-4.5 were pooled and rechromatographed in a silica gel column in the isocratic mode with Hex-EtOAc (8:2) to form **Mt-1** (21 mg) and a mixture of **Mt-1**, **Mt-2** and **Mt-3**, which was named **Mixture 1** (102 mg). The HF-6 and HF-7 fractions were pooled and rechromatographed in successive silica gel columns with different mobile phases: Hex-EtOAc (8:2), Hex-CH_2_Cl_2_-MeOH (10:10:1) and hex-EtOAc-petroleum ether (6:2:2), which resulted in **Mt-3** (32 mg) and **Mt-4** (19 mg).

The DF (2.0 g) was fractionated by column chromatography with silica gel (1:40) eluted with Hex-EtOAc (95:5, 9:1, 8:2, 7:3, 6:4, 1:1), EtOAc 100% and EtOAc-MeOH (1:1). Fifty-nine 12 mL fractions were collected and evaluated by TLC (hexane-EtOAc (10–30%)), observed under UV light, and detected with sulphuric vanillin reagent, which resulted in 17 new fractions (DF-1 a DF-17). Fractions DF-7 and DF-8 were united and rechromatographed in a silica gel column with petroleum ether-AcOEt (8:2), and 25 fractions of 3 mL each were collected. According to the TLC chromatographic profile, these fractions were collected in seven new fractions (DF-7.1 to DF-7.7). The DF-7.2 fraction was isocratically rechromatographed with Hex-AcOEt (8:2), which resulted in **Mt-1** (199 mg), a mixture of **Mt-1** and **Mt-2** named **Mixture 2** (86 mg) and a mixture of **Mt-5** and **Mt-6** named **Mixture 3** (12 mg). The DF-10 fraction was rechromatographed in a silica gel column with CH_2_Cl_2_-AcOEt-petroleum ether (6:2:2). The obtained substance from this column was recrystallized from methanol and resulted again in **Mt-4** (29 mg).

The EAF was fractionated by column chromatography with silica gel (1:40). In two independent initial columns, similar fractions of these columns were pooled to prepare for the Sepacore preparative chromatograph. In the first column, the EAF (9.0 g) was eluted with CH_2_Cl_2_-AcOEt (9:1, 8:2, 7:3, 6:4, 1:1, 4:6, 3:7, 1:9), AcOEt (100%), AcOEt-MeOH (8:2, 1:1, 2:8) and MeOH (100%). In total, 91 fractions were collected with aliquots of 30 mL for fractions 1–50 and 10 mL for fractions 51–91. The fractions were analyzed by TLC using AcOEt-formic acid-acetic acid-H_2_O (100:11:11:26) as the mobile phase. Based on *R*_f_ of the spots observed in UV light at 254/365 nm and revealed with NP, the fractions were pooled into 11 new fractions (EA-1 to EA-11). The combined fractions were dissolved in methanol at a ratio of 1 mg/mL and analyzed using analytical HPLC for a better fraction analysis. Thus, fractions EA-2 and EA-4 were referred to ^1^H-NMR and resulted in **Mt-7** (13 mg) and a mixture of **Mt-7** and **Mt-8** named **Mixture 4** (35 mg), respectively. Fractions EA-7 to EA-11 were pooled and rechromatographed in a silica gel column eluted with AcOEt (100%), AcOEt-MeOH (9:1, 7:3, 1:1, 3:7) and MeOH (100%), which resulted in **Mt-9** (183 mg). Fractions EA-7.3 to EA-7.5 were analyzed in analytical HPLC, pooled with similar fractions from the second column and submitted to the Sepacore preparative chromatograph, as later described. In the second column, the EAF (12.0 g) was eluted with CH_2_Cl_2_-AcOEt (2:8), AcOEt (100%), AcOEt-MeOH (9:1, 8:2, 7:3, 6:4, 1:1, 4:6, 2:8) and MeOH (100%). In total, 72 fractions were collected with aliquots of 30 mL for fractions 1–42, 60 mL for fractions 43–70 and 200 mL for fractions 71–72. The fractions were analyzed by TLC and collected in 11 new fractions (N4-1 to N4-11). The pooled fractions were analyzed in analytical HPLC and again resulted in **Mt-9** (79.3 mg). Fractions N4-1 and N4-2 were pooled and submitted to the Sepacore preparative chromatograph with a C-18 column. Then, they were eluted with H_2_O-MeOH (30, 40, 50, 70% and 100%) MeOH to form **Mt-9** (4.5 mg) and **Mt-10** (2.9 mg). Finally, fractions N4-4 and N4-5 were pooled with fractions EA-7.3 to EA-7.5 and subjected to five chromatographic steps in C-18, which was eluted with H_2_O-MeOH (10, 20, 30, 40, 50, 70%) and 100% MeOH, to yield **Mt-11** (30.5 mg).

### 3.4. Microbial Strains

The studied microorganisms are as follows: reference strains of *Staphylococcus aureus*, *Staphylococcus epidermidis*, *Micrococcus roseus*, *Micrococcus luteus*, *Bacillus cereus*, *Bacillus subtilis*, *Escherichia coli*, *Enterobacter cloacae*, Enterobacteraerogenes, Enterobacteraerogenes, *Pseudomonas aeruginosa*, *Serratia marcescens*, *Salmonella* sp., *Candida albicans*, *Candida parapsilosis* and *Cryptococcus neoformans* species complex, obtained from the American Type Culture Collection (ATCC) standard strains, except of theclinical isolates of *Pseudomonas aeruginosa* (SPM1), *C. albicans* (2, 3, 48, 111, 138, 181), *C. parapsilosis* (11) and *C. neoformans* species complex (L1, L2), which belong to the collection of the Laboratory of Medical Bacteriology and Laboratory of Mycology at the Institute of Tropical Pathology and Public Health from Federal University of Goiás, Goiânia, GO, Brazil (IPTSP-UFG). They were maintained at 4 °C. Prior to testing, the bacteria were transferred to Casoy agar (Difco) and incubated at 37 °C for 24 h; while the fungi were transferred to Sabouraud agar (Difco) and incubated at room temperature for 24–48 h for *Candida* spp. and 48–72 h for the *C. neoformans* species complex.

### 3.5. In Vitro Susceptibility Testing

The in vitro activity of the ethanolic leaf extract, their fractions and compounds of *M. tomentosa* was evaluated using the broth microdilution method, as described in the Clinical and Laboratory Standards Institute (CLSI) documents M07-A8 for bacteria and M27-A3 and M27-S4 for yeasts [43,44,45].

In the antibacterial test, 200 μL of plant extract in initial concentration of 2000 μg/mL was diluted in Mueller-Hinton broth and 10% dimethyl sulfoxide (DMSO). Serial two-fold dilutions were conducted in 96-well microplates for the final concentrations of 1000 to 1.95 μg/mL of the extract or fractions. A volume of 5 μL containing 10^4^ UFC/mL of microbial inoculum was added to each well plate and incubated at 35 °C for 18–20 h. The bacterial growth was visualized by adding 0.5% triphenyl tetrazolium chloride to each well and analyzed after 30 min of incubation. The appearance of reddish color was considered as proof of bacterial growth.

In the antifungal activity, the samples were diluted in an RPMI 1640 medium with l-glutamine without bicarbonate, which was then buffered with 0.165 M MOPS (morpholine propane sulfonic acid) and 5–10% DMSO. Serial two-fold dilutions were conducted in 96-well microplates for the final concentrations of 1024 to 1 μg/mL of the extract or fractions and 128 to 0.125 μg/mL for each compound. A volume of 100 μL of microbial inoculum at a concentration of 10^3^ UFC/mL was added to each well and incubated at 35 °C for 48 h for *Candida* sp. and at room temperature for 72 h for the *Cneoformans* species complex. Fungal growth was checked visually and the MIC was defined as the lowest concentration, which resulted in total inhibition of visible microorganism growth.

The tests were performed in duplicatein two independent experiments. Vancomycin (Sigma-Aldrich, St. Louis, MO, USA) and gentamicin (Sigma-Aldrich, St. Louis, MO, USA) were used as controls for Gram-positive and Gram-negative bacteria, respectively; fluconazole (Sigma-Aldrich, St. Louis, MO, USA) and itraconazole (Sigma-Aldrich, St. Louis, MO, USA) were used as controls for *Candida* spp. and the *Cryptococcus neoformans* species complex, respectively.

### 3.6. Structural Elucidation

*α-bisabolol* (**Mt-1**): ^1^H-NMR (500 MHz, CDCl_3_): 1.11 (s; H-7’); 1.49 (m; H-3’); 1.58 (s; H-1); 1.62 (s; H-8’); 1.65 (s; H-7); 1.69 (s; H-1’); δ 5.13 (t, *J* = 7.2 Hz; H-5’,); 5.37 (m; H-3). ^13^C-NMR (125 MHz, CDCl_3_): 17.71 (C8’); 22.02 (C4’); 23.22 (C6); 23.32 (C7/C7’), 25.66 (C1’); 26.97 (C2); 31.12 (C5); 40.21 (C3’); 43.14 (C1); 73.94 (C2’); 120.47 (C3); 124.73 (C5’); 131.58 (C6’); 134.16 (C4).

*β-sitosterol* (**Mt-4**): ^1^H-NMR (500 MHz, CDCl_3_): 0.68 (s; H18); 0.81(d; 6.8; H26); 0.83 (d; 6.9; H27); 0.85 (t; 7.4; H29); 0.92 (d; 6.5; H21); 1.01(s; H19); 3.53 (sept; 4.3; H3); 5.35 (m; H6). ^13^C-NMR (125 MHz, CDCl_3_): 11.90 (C29);12.0 (C18); 18.67 (C26); 19.21 (C19); 19.30 (C21); 19.84 (C27); 20.25 (C11); 22.90 (C28); 25.84 (C15); 26.05 (C25); 28.16 (C16); 31.31 (C2); 31.84 (C7/C8); 33.90 (C22); 36.05 (C20); 36.27 (C10); 36.51 (C1); 39.20 (C23); 39.75 (C12); 42.16 (C13); 42.51 (C4); 45.63 (C24); 49.73 (C9); 56.04 (C17); 56.33 (C14); 71.82 (C3); 121.79 (C6); 140.73 (C5).

*Quercetin* (**Mt-7**): ^1^H-NMR (500 MHz, CD_3_OD): 6.18 (d; 1.9; H6); 6.39 (d; 2.1; H8); 6.88(d; 8.4; H5’); 7.61 (dd; 2.1; 8.4; H6’); 7.73 (d; 2.1; H2’). ^13^C-NMR (125 MHz, CD_3_OD): 93.1 (C8); 97.8 (C6); 102.7 (C10); 114.0 (C3’); 114.6 (C2’); 114.7 (C5’); 120.4 (C6’); 122.2 (C1’); 137.8 (C3); 147.1 (C2); 147.6 (C4’); 156.6 (C9); 163.4 (C5); 166.1 (C7); 175.3 (C4).

*Kaempferol* (**Mt-8**): ^1^H-NMR (500 MHz, CD_3_OD): 6.18 (d; 1.9; H6); 6.40 (d; 1.9; H8); 6.91 (d; 8.9; H3’/H5’); 8.09 (d; 8.9; H2’/H6’). ^13^C-NMR (125 MHz, CD_3_OD): 92.8 (C8); 97.8 (C6); 103.7 (C10); 114.1 (C3’/C5’); 120.3 (C1’); 129.4 (C2’/C6’); 133.4 (C3); 156.3 (C2); 156.3 (C9); 159.1 (C4’); 161.0 (C5); 164.3 (C7); 179.2 (C4).

*Quercetin-3-O-α-arabinofuranose (avicularin)* (**Mt-9**): ^1^H-NMR (500 MHz, CD_3_OD): 3.51 (d; 4.4; H5’’); 3.87 (t; 4.2; H4’’); 3.91 (m; H3’’); 4.33 (dd; 0.9; 2.9; H2’’); 5.47 (brs; H1’’); 6.21 (d; 2.1; H6); 6.40 (d; 2.1; H8); 6.90 (d; 8.4; H5’); 7.49 (dd; 2.1; 8.4; H6’); 7.53(d; 2.1; H2’). ^13^C-NMR (125 MHz, CD_3_OD): 61.2 (C5’’); 77.3 (C3’’); 82.1 (C2’’); 86.6 (C4’’); 93.4 (C8); 98.5 (C6); 104.1 (C10); 108.4 (C1’’); 114.9 (C5’); 115.4 (C2’); 121.7 (C6’); 138.2 (C3); 144.9 (C3’); 148.5 (C4’); 158.1 (C2); 159.7 (C5).

*Kaempferol-3-O-α-arabinofuranose (juglanin)* (**Mt-10**): ^1^H-NMR (500 MHz, CD_3_OD): 3.48 (m; H5’’); 3.80 (m; H4’’); 3.90 (m; H3’’); 4.31 (dd; 1.2; 3.2; H2’’); 5.49 (s; H1’’); 6.22 (d; 2.0; H6); 6.41(d; 2.0; H8); 6.92 (d; 8.7; H3’/5’); 7.96 (d; 8.7; H2’/6’). ^13^C-NMR (125 MHz, CD_3_OD): 60.9 (C5’’); 77.0 (C3’’); 81.7 (C2’’); 86.7 (C4’’); 93.4 (C8); 98.5 (C6); 108.2 (C1’’); 114.9 (C3’/5’); 121.3 (C1’); 130.3 (C2’/6’); 159.8 (C4’). 

*Quercetin-3-O-α-arabinopyranoside (guaijaverin)* (**Mt-11**): ^1^H-NMR (500 MHz, CD_3_OD): 3.44 (d; 2.0; H5’’); 3.66 (dd; 3.2; 8.3; H3’’); 3.82 (m; H4’’); 3.90 (m; H2’’); 5.16 (d; 6.4; H1’’); 6.18 (d; 2.0; H6); 6.37 (d; 2.0; H8); 6.87 (d; 8.7; H5’); 7.56 (dd; 2.4; 8.7; H6’); 7.75 (d; 2.4; H2’). ^13^C-NMR (125 MHz, CD_3_OD): 67.0 (C5’’);68.6 (C4’’); 73.0 (C2’’); 73.9 (C3’’); 94.6 (C8); 99.9 (C6); 103.9 (C10); 104.7 (C1’’); 116.2 (C5’); 117.0 (C2’); 123.1 (C6’); 134.7 (C3); 144.5 (C3’); 148.7 (C4’); 157.3 (C2); 157.3 (C9); 161.6 (C5); 165.1 (C7).

## Figures and Tables

**Table 1:** Minimal inhibitory concentration (μg/mL) of the crude extract and fractions from the *Myrcia tomentosa* leaves and reference antimicrobials against bacteria and yeasts.

Microorganisms	EB	HF	DF	EAF	AF	Vanc.	Genta.	Fluc.	Itrac.
Bacteria									
*Bacillus cereus* ATCC 14579	1000	250	250	1000	1000	2			
*Bacillus subtilis* ATCC 6633	1000	500	500	1000	1000	0.25			
*Micrococcus luteus* ATCC 9341	500	500	500	1000	1000	0.25			
*Micrococcus roseus* ATCC 1740	500	500	500	1000	1000	0.5			
*Staphylococcus aureus* ATCC 25923	750	500	500	1000	1000	1			
*Staphylococcus aureus* ATCC 6538	1000	250	>1000	1000	1000	2			
*Staphylococcus epidermidis* ATCC 12229	1000	250	250	1000	1000	1			
*Enterobacter aerogenes* ATCC 13048	>1000	>1000	>1000	>1000	>1000		0.125		
*Enterobacter cloacae* HMA/FTA502	>1000	>1000	>1000	>1000	>1000		4		
*Escherichia coli* ATCC 11229	>1000	>1000	>1000	>1000	>1000		2		
*Escherichia coli* ATCC 8739	>1000	1000	>1000	>1000	>1000		8		
*Pseudomonas aeruginosa* ATCC 27483	500	1000	>1000	500	250		4		
*Pseudomonas aeruginosa* SPM1	500	1000	1000	250	125		4		
*Salmonella sp* ATCC 19430	1000	>1000	>1000	1000	1000		2		
*Serratia marcescens* ATCC 14756	>1000	>1000	>1000	>1000	>1000		4		
Yeasts									
*Candida albicans* ATCC 90028	32	128	128	32	32			1	
*Candida albicans* 02	32	64	128	16	16			2	
*Candida albicans* 03	4	32	64	8	8			>64	
*Candida albicans* 48	16	64	256	16	32			>64	
*Candida albicans* 111	8	128	16	16	16			>64	
*Candida albicans* 138	8	32	32	4	4			8	
*Candida albicans* 181	8	64	128	16	16			>64	
*Candida parapsilosis* ATCC 22019	16	64	64	16	16			1	
*Candida parapsilosis* 11	16	64	64	16	16			1	
*Cryptococcus neoformans* ATCC 28957	16	16	32	>1024	>1024				2
*Cryptococcus gatti* L1	16	16	32	1024	1024				2
*Cryptococcus neoformans* L2	16	64	16	1024	1024				2

EB: crude ethanol extract. HF: hexane fraction. DF: dichloromethane fraction. EAF: ethyl acetate fraction. AF: aqueos fraction. Vanc.: vancomycin. Gent.: gentamicin. Fluc.: fluconazole. Itrac.: itraconazole.

**Table 2:** Minimum inhibitory concentration (μg/mL) of the flavonoids isolated from the leaves of *M. tomentosa*.

Yeasts	Quercetin	Quercetin + Kaempferol	Avicularin	Juglanin	Guaijaverin
*C. albicans*					
ATCC 90028	>128	>128	16	>128	>128
02	64	128	4	128	16
03	>128	>128	8	>128	>128
48	>128	>128	8	>128	>128
111	>128	>128	8	>128	128
138	128	>128	2	16	2
181	>128	>128	4	>128	>128
*C. parapsilosis*					
ATCC 22019	>128	>128	32	128	16
11	64	128	8	64	8
